# Metabolic Rewiring toward Oxidative Phosphorylation Disrupts Intrinsic Resistance to Ferroptosis of the Colon Adenocarcinoma Cells

**DOI:** 10.3390/antiox11122412

**Published:** 2022-12-06

**Authors:** Célia Gotorbe, Jérôme Durivault, Willian Meira, Shamir Cassim, Maša Ždralević, Jacques Pouysségur, Milica Vučetić

**Affiliations:** 1 Medical Biology Department, Centre Scientifique de Monaco (CSM), 98000 Monaco, Monaco; 2 Centre A. Lacassagne, University Côte d’Azur, Institute for Research on Cancer & Aging (IRCAN), CNRS, INSERM, 06100 Nice, France

**Keywords:** ferroptosis, glutathione peroxidase 4, ferroptosis suppressor protein 1, colorectal adenocarcinoma, Warburg effect, OXPHOS

## Abstract

Glutathione peroxidase 4 (GPX4) has been reported as one of the major targets for ferroptosis induction, due to its pivotal role in lipid hydroperoxide removal. However, recent studies pointed toward alternative antioxidant systems in this context, such as the Coenzyme Q-FSP1 pathway. To investigate how effective these alternative pathways are in different cellular contexts, we used human colon adenocarcinoma (CRC) cells, highly resistant to GPX4 inhibition. Data obtained in the study showed that simultaneous pharmacological inhibition of GPX4 and FSP1 strongly compromised the survival of the CRC cells, which was prevented by the ferroptosis inhibitor, ferrostatin-1. Nonetheless, this could not be phenocopied by genetic deletion of FSP1, suggesting the development of resistance to ferroptosis in FSP1-KO CRC cells. Considering that CRC cells are highly glycolytic, we used CRC Warburg-incompetent cells, to investigate the role metabolism plays in this phenomenon. Indeed, the sensitivity to inhibition of both anti-ferroptotic axes (GPx4 and FSP1) was fully revealed in these cells, showing typical features of ferroptosis. Collectively, data indicate that two independent anti-ferroptotic pathways (GPX4-GSH and CoQ10-FSP1) operate within the overall physiological context of cancer cells and in some instances, their inhibition should be coupled with other metabolic modulators, such as inhibitors of glycolysis/Warburg effect.

## 1. Introduction

Ferroptosis is a newly described type of iron-dependent cell death caused by the uncontrolled accumulation of reactive oxygen species (ROS) that leads to specific oxidative damage of polyunsaturated fatty acids (PUFAs) in the plasma membrane, and thus, to loss of its integrity and selective permeability [1]. The selenoenzyme glutathione peroxidase 4 (GPX4) plays a central role in protecting membrane PUFAs from these oxidative insults, i.e., in protecting cells from ferroptosis by converting lipid hydroperoxides into their less toxic alcohol derivatives. To do so, GPX4 uses the reducing power of glutathione (GSH) or other small cellular thiols [2,3,4,5], which explains why inhibition of GPX4 or deprivation of GSH or cysteine are the most frequently reported conditions triggering ferroptosis [1,4,6,7,8].

The extensive body of literature accumulated over the last decade unequivocally indicates the great potential of ferroptosis as a strategy for the eradication of tumor cells. During this period, our team, focusing primarily on highly aggressive pancreatic ductal adenocarcinoma (PDAC), has dissected in detail the pivotal antioxidant axis: cysteine-GSH-GPX4, used by cancer cells to forestall ferroptosis [4,8,9,10,11,12]. Our work and that of others have shown that the most potent and effective way to induce ferroptosis in PDAC and other types of cancers is either to block cysteine import into the cell, by inhibiting the membrane transporter known as xCT or to block the activity of GPX4 enzyme (reviewed in [13]). Although these strategies hold great promise for the development of novel and more effective anticancer therapeutics, it is necessary to understand and foresee potential escape routes cancer calls might employ to evade ferroptosis.

GPX4 has long been seen as a unique enzyme responsible for the removal of lipid hydroperoxides [2,6]. Nonetheless, two independent studies from 2019 revealed yet another and alternative antioxidant route, which involves ubiquinol, more commonly known as Coenzyme Q10 (CoQ10) [14,15]. CoQ10 is a lipophilic antioxidant detected at high levels in mitochondrial and non-mitochondrial compartments, including the plasma membrane [16,17]. Outside of mitochondria, once used, CoQ10 can be regenerated by the NADH-dependent CoQ oxidoreductase function of Ferroptosis Suppressor Protein 1 (FSP1) [18]. FSP1 is localized in the plasma membrane due to its canonical myristylation motif, making this pathway directly adjacent to lipid hydroperoxides [14]. The individual importance of these two pathways (GSH-GPx4 and CoQ10-FSP1) for the wide range of tumor types, especially those intrinsically resistant to conventionally used ferroptosis inducers, still have not been entirely clarified.

Besides alternative antioxidative pathways, the metabolic plasticity of the cancer cells, which regulates intracellular ROS production has been indicated as a potential player in ferroptosis sensitivity [19,20]. Mitochondria and oxidative metabolism are the major sources of cellular ROS production. Rapidly growing, and proliferating cancer cells require a high metabolic rate, which renders them intrinsically oxidatively compromised [21]. However, by switching from OXPHOS to fermentative glycolysis, cancer cells, at least in part, relieve this ROS stress that otherwise would be overwhelming if they would rely exclusively on the OXPHOS for growth and proliferation. Consistently, it has been observed that tumor cells undergoing ferroptosis showed substantially lower glycolytic activities [22,23], which potentially could be explained by metabolic rewiring to OXPHOS and consequent elevation in the cellular ROS levels. However, this hypothesis has not been fully investigated up to know.

In the present study, we used colorectal adenocarcinoma (CRC) cells which show high intrinsic resistance to ferroptosis (LS174T, HCT-15, HCT-116 and SW480). Data obtained in the study indicate that pharmacological inhibition of both anti-ferroptotic enzymes GPX4 and FSP1 is necessary to sensitize CRC cells to ferroptosis. Interestingly, however, genetic deletion of FSP1 seems to induce adaptation of CRC cells to GPX4 inhibition, which was completely prevented by rewiring cellular metabolism toward OXPHOS. Another important observation of the study was that Warburg-null cells show much higher resistance to GPX4 inhibition in comparison with their WT counterparts, most likely due to their slower growth/metabolic rate.

## 2. Material and Methods

### 2.1. Cell Culture 

Human colon adenocarcinoma LS174T (kindly provided by Dr. Van de Wetering), HCT-15, HCT-116, SW480 (kindly provided by Dr. Anne-Odile Hueber) and mouse B16-F10 (ATCC^®^ CRL-6475™; ATCC, Manassas, VA, USA) were cultivated at 37 °C and 5% CO_2_ in DMEM (Gibco, Gaithersburg, MD, USA) supplemented with 7.5% FBS, penicillin (10 U/mL) and streptomycin (10 µg/mL). The glycolysis-null cells (LDHA/B-double knockout) were obtained and described previously by [24].

The cells have been authenticated and routinely tested for Mycoplasm (PlasmoTest Mycoplasma Detection Kit; InvivoGen, San Diego, CA, USA). Experiments were conducted in the same media supplemented with RSL3 (Sigma-Aldrich, St. Louis, MO, USA, SML2234) and/or Ferrostatin-1 (Fer-1) (Sigma-Aldrich, St. Louis, MO, USA, SML0585) or in DMEM without cysteine (Gibco, Waltham, MA, USA, 21013-024) supplemented with methionine, glutamine and 7.5% dialyzed serum.

### 2.2. Genomic Disruption of FSP1 Using CRISPR-Cas9

LS174T and B16-F10 cells with wild-type (WT) background or with genetic deletion of lactate dehydrogenase A and B (LDHA/B-DKO) were transfected with pSpCas9(BB)-2A-GFP (PX458) plasmids containing CRISPR-Cas9 targeting regions of first and second exon of the FSP1 gene using Nucleofection (Lonza, Bale, Switzerland). As the PX458 plasmid contains GFP, single-cell sorting was conducted by FACS analysis (BD FACSMelody^TM^, Allschwil, Switzerland) after 24 h post-transfection, and seeded in 96-well plates containing DMEM supplemented with 7.5% FBS and 1 mM NAC (Sigma-Aldrich, St. Louis, MO, USA) + 2 µM Fer-1. Screening of the clones via Western blot assessed the validation of the FSP-KO clones and two independent KO clones were chosen for subsequent experiments, to minimize the clonal effect.

### 2.3. Clonogenicity Assay

The different cell lines (1000 cells per dish) were seeded in 60 mm dishes and incubated at 37 °C, 5% CO_2_ with normal DMEM media supplemented with 7.5% FBS (control condition) or in presence of different concentrations of RSL3 (treated condition), with or without 4 µM Fer-1 (rescue condition). After 7 (for B16-F10) or 14 days (for LS174T) dishes were stained with 5% Giemsa (Sigma Aldrich, St. Louis, MO, USA, 48900) for 30 to 45 min to visualize colonies.

### 2.4. Immunoblotting

Cells were lysed in 1.5× Laemmli buffer, and protein concentrations were determined using BCA protein assay (Thermo Fisher Scientific, Waltham, MA, USA). Protein extracts (15–20 µg) were separated by electrophoresis on 10 or 12% SDS-polyacrylamide gel and transferred onto polyvinylidene difluoride membranes (Immobilon, Merck Milipore Ltd., Tullagen, Carrigtwohill, County Cork, Ireland). Membranes were blocked in 5% non-fat milk in PBS and incubated with anti-human or anti-mouse primary antibodies against xCT (Cell Signaling, Danvers, MA, USA, 126915), GPX4 (Abcam, Cambridge, UK, ab125066) and FSP1 (Assay biotech, C12047). The protein loading control was verified by the detection of Tubulin (Cell Signaling Technology, Danvers, MA, USA, 2128) and HSP90 (Thermo Scientific, Waltham, MA, USA, MA1-10379).

Immunoreactive bands were detected with horseradish peroxidase anti-mouse or anti-rabbit antibodies (Promega, Medison, WI, USA) using the ECL system (Merck Milipore, Watford, UK). Immunoblot analysis was performed using the Li-COR Odyssey Imaging System (Lincoln, NE, USA).

### 2.5. FACS Analysis

FACS analysis of cell death and lipid hydroperoxides accumulation were performed at least three times, 10,000 cell events were analyzed per sample using a BD FACS Melody cytometer and data were analyzed using FlowJo software version vX.0.7 (Ashland, OR, USA).

#### 2.5.1. Cell Death

Cells were seeded in 6-well dishes and collected by trypsinization, merged with the corresponding supernatant, after 24 h or 48 h of treatment with different concentrations of RSL3 and rescue condition by addition of 4 µM Fer-1. Following centrifugation, the cells were re-suspended in FACS buffer (PBS, 0.2% BSA, 2 mM EDTA) and stained, just before the analysis, with 2 µg/mL PI (Invitrogen, Waltham, MA, USA).

#### 2.5.2. Detection of Lipid Hydroperoxides

The cells were seeded in 6-well dishes with the associated treatment and on the day of analysis, incubated with 2 µM BODIPY 581/591 C11 (Molecular Probes, Eugene, OR, USA) for 30 min at 37 °C/5% CO_2_ protected from the light. Following, the cells were washed two times with PBS, collected with accutase (Dutsher, Bernolsheim, France) and resuspended in FACS buffer (PBS, 0.2% BSA, 2 mM EDTA). The data are represented in modal scaling (each peak is normalized to its mode, i.e., to % of the maximal number of cells found in a particular bin).

#### 2.5.3. ROS Level

Cells were seeded in 6-well dishes and on the day of analysis, trypsinized and incubated with 2 µM DCFDA (Abcam, Cambridge, UK, ab113851) for 30 min at 37 °C/5% CO_2_ protected from the light. Following, FACS analysis of total ROS level was performed, and the data are represented in modal scaling (each peak is normalized to its modal, i.e., to % of the maximal number of cells found in a particular bin).

### 2.6. Statistical Analysis

Data are expressed as mean ± SEM. Each experiment was performed at least three times. Statistical analysis was conducted with an unpaired Student’s *t*-test. Differences between groups were considered statistically significant when *p* < 0.05 (*), *p* < 0.01 (**), and *p* < 0.001 (***).

## 3. Results

### 3.1. Colorectal Adenocarcinoma (CRC) Cell Lines Show Great Resistance to Ferroptosis Induction

**Figure 1:**
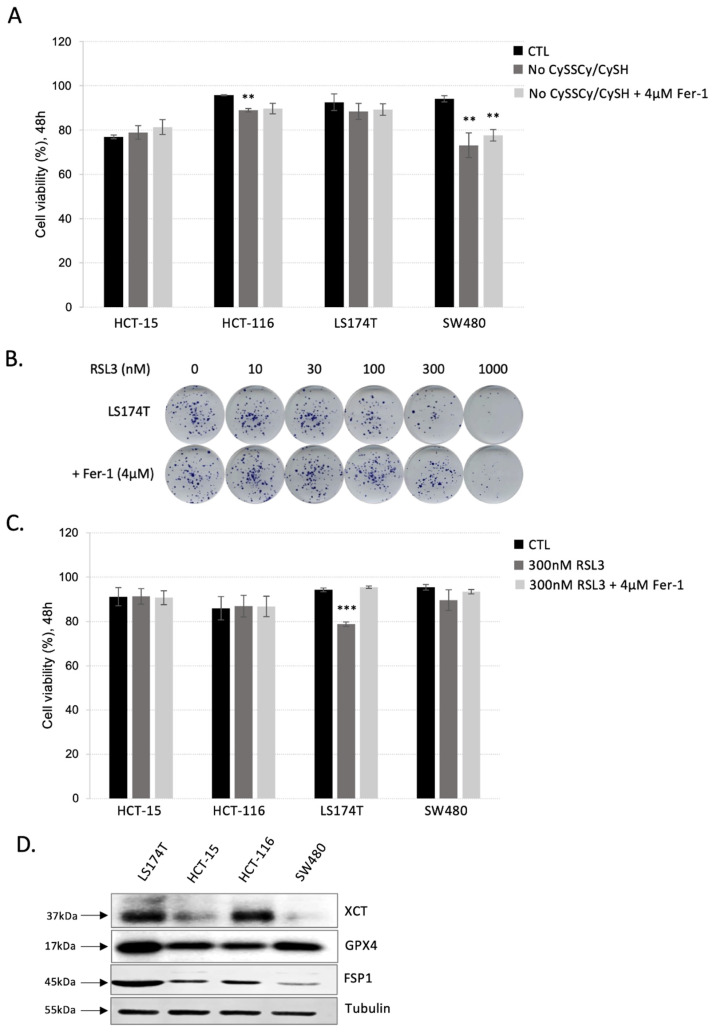
CRC cell lines are resistant to ferroptosis induced by CySSCy/CySH starvation or GPX4 inhibition. (**A**) Different CRC cell lines (HCT-15, HCT-116, SW480, LS174T) were seeded in media with or without cyst(e)ine, supplemented or not with 4 µM Ferrostatin-1 (Fer-1), and cell viability was analyzed after 48 h. The results are presented as mean ± SEM, *n* = 3. ** *p* < 0.01, comparison with corresponding cell line in control conditions. (**B**) LS174T cells were cultivated in DMEM media supplemented with increasing concentration of GPX4 inhibitor—RSL3 (0, 10, 30, 100, 300, 1000 nM), supplemented or not with 4 µM Ferrostatin-1 (Fer-1). After 14 days colonies were colored for visualization using Giemsa. Representative images are shown. (**C**) Different CRC cell lines (HCT-15, HCT-116, SW480, LS174T) were seeded in media supplemented or not with 300 nM RSL3 in the presence or not of 4 µM Ferrostatin-1 (Fer-1). Cell viability was analyzed after 48 h. The results are presented as mean ± SEM, *n* = 3. *** *p* < 0.001, comparison with corresponding cell line in control conditions. (**D**) Protein contents of the major players involved in ferroptosis prevention: glutathione peroxidase 4 (GPX4), cystine-glutamate exchanger (xCT) and ferroptosis suppressor protein 1 (FSP1) were analyzed by Western blot in 4 different CRC cell lines seeded in standard DMEM media for 24 h. Blots are representative of three independent experiments.

**Figure 2:**
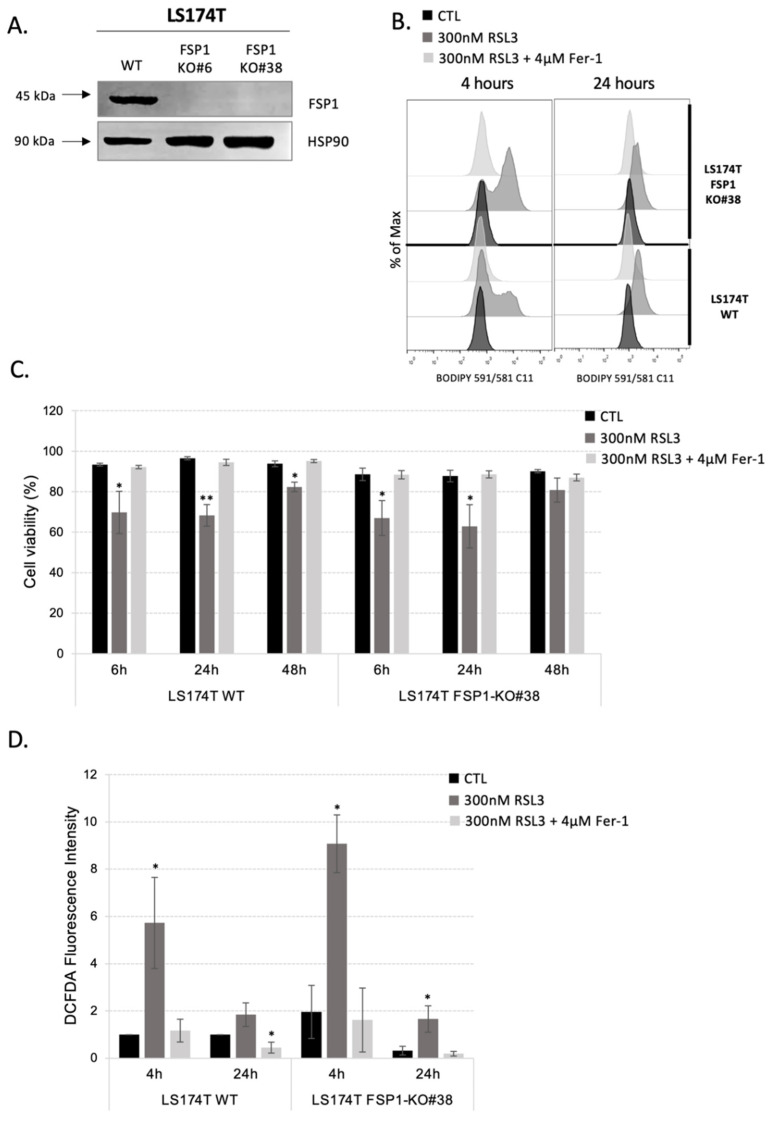
Pharmacological inhibition of GPX4 in the LS174T cells with FSP1-KO background is not sufficient to induce ferroptosis. (**A**) The expression of FSP1 was analyzed in LS174T WT and two independent FSP1-KO (#6 and #38) clones. Three independent experiments were performed, and representative blots are shown. (**B**) Lipid hydroperoxide content was measured in LS174T WT and FSP1-KO#38 cells 4 h and 24 h upon seeding in media supplemented or not with 300 nM RSL3 and 4 µM Fer-1. Presented histograms are representative of three independent experiments. (**C**) LS174T WT and FSP1-KO#38 cells were seeded in media supplemented or not with 300 nM, in the presence or not of 4 µM Fer-1. Cell viability was analyzed after 6 h, 24 h and 48 h. The results are presented as mean ± SEM, *n* = 3. * *p* < 0.05, ** *p* < 0.01, comparison with corresponding cell line in control conditions. (**D**) Intracellular ROS level was measured by DCFDA staining (see Section 2) in LS174T WT and FSP1-KO#38 4 h and 24 h after seeding in media supplemented or not with 300 nM RSL3 and 4 µM Fer-1. Intracellular ROS levels are presented in fold change (mean ± SEM, *n* = 3). * *p* < 0.05, comparison with corresponding cell line in control conditions.

To investigate the sensitivity of CRC cells to ferroptosis, cell death of LS174T, HCT-15, HCT-116 and SW480 cultured in media supplemented or not with cysteine. According to the data, all four cell lines showed resistance to cysteine starvation during 48 h (Figure 1A). A very modest, but statistically significant effect was observed in the case of LS174T and HCT-116 cell lines. Nonetheless, this effect was not reverted by the addition of ferroptosis inhibitor—ferrostatin-1 (Fer-1). Next, the sensitivity of CRC cell lines to ferroptosis was investigated also by using the inhibitor of the major antioxidant enzyme, GPX4, involved in the removal of lipid hydroperoxides (RSL3). Increasing the dose of the RSL3 inhibitor had a very modest effect on the clonogenicity potential of the LS174T cells up to 1000 nM concertation of the drug (Figure 2B). Furthermore, the effects observed with 1000 nM RSL3 were not reverted by ferrostatin-1, suggesting off-target effects of the inhibitor at the highest investigated concentration. As expected, the lower dose (300 nM) had no effect on the cell survival of either CRC cell line grown in 2D within a 48 h time range (Figure 1C). Again, the modest exception was observed in the case of SW480 cells, and this effect was reverted by the addition of ferrostatin-1, suggesting that this CRC cell line might be slightly more sensitive to ferroptosis by GPX4 inhibition in comparison with LS174T, HCT-116 and HCT-15.

To further understand ferroptosis resistance in CRC, we analyzed the protein content of the major proteins involved in its prevention. As shown in Figure 1D, the major actors of the two ferroptosis-preventing pathways—xCT, GPX4 and FSP1, were abundantly expressed in LS174T when compared with other CRC cell lines. Similarly, high content of the xCT was detected in HCT-116, while FSP1 and GPX4 expression levels were about in the same range in HCT-15, HCT-116 and SW480.

### 3.2. Inhibition of Both Ferroptosis-Preventing Pathways, GSH-GPX4 and FSP1-CoQ10, Did Not Sensitize CRC to Ferroptosis

The cysteine-GSH-GPX4 axis initially has been seen as the only ferroptosis-preventing pathway. In 2019, two independent studies published in Nature revealed the importance of yet another route for the removal of lipid hydroperoxides and ferroptosis prevention [14,15]. This involves reduced CoQ10 and its regenerating enzyme, known as FSP1. Considering that CRC cells showed resistance to GPX4 inhibition, we hypothesized that this alternative pathway may be involved in ferroptosis prevention in these cells. To investigate this issue, we used a selective and glutathione-independent inhibitor of FSP1 named iFSP1. Treatment of 24 h with iFSP1 inhibitor in LS174T and SW480 did not impact the survival of these cells. However, the combination of GPx4 inhibition by RSL3 inhibitor and FSP1 inhibition by iFSP1 inhibitor was able to sensitize CRC to ferroptosis by inducing approximately 50 and 60% cell death in LS174T and SW480, respectively. The observed effect was completely rescued by the addition of ferrostatin-1 (Supplementary Figure S1).

Considering that major actors of ferroptosis prevention are highly expressed in LS174T, we chose this cell line to generate FSP1-KO cells using CRISPR-Cas9 technology with the aim of better understanding the synergic action of GPX4 and FSP1. Genomic DNA analysis and sequencing of the CRISPR-targeted site revealed disruptive mutations in FSP1 that resulted in the lack of the corresponding protein expression in FSP1-KO clones, as shown by Western blot analysis (Figure 2A). Two independent clones were analyzed and showed the same phenotype, and for convenience, all the results obtained with one of the clones are shown in the Supplementary Materials.

One of the most important molecular markers of ferroptosis is the accumulation of oxidative damage in membrane lipids. We investigated, the level of lipid hydroperoxides in LS174T WT and FSP1-KO cells in the control conditions and after RSL3 treatment for 4 h and 24 h (Figure 2B and Supplementary Figure S2A) and we related these results to cell death analysis after 6 h, 24 h and 48 h (Figure 2C and Supplementary Figure S2B). Data presented in Figure 2B,C, unequivocally showed that genetic deletion of FSP1 protein alone does not alter lipid hydroperoxide profile or viability of the cells within 48 h time range. In line with our hypothesis, the addition of an RSL3 inhibitor to the media increased lipid hydroperoxide content more prominently in the FSP1-KO cells in comparison with their WT counterparts. Surprisingly, this difference was not translated into the survival difference as seen previously with the pharmacological approach. Additionally, the treatment of FSP1-KO cells with iFSP1 inhibitor did not induce cell death after 24 h, suggesting no off-target effect of the pharmacological approach (Supplementary Figure S2C).

Namely, although FSP1-KO cells initially (after 4 h) accumulated more oxidative damage in the lipid compartment, approximately 30% of cell death was equally detected in both FSP1-KO and WT cells after 6 h and 24 h. Very interesting to us was the fact that after 24 h almost no detectable increase in lipid hydroperoxide content was observed in both WT and FSP1-KO cells (Figure 2B and Supplementary Figure S2A). Even more, after 48 h of RSL3 treatment only very modest cell death was detectable in both cell lines, reaching statistical significance surprisingly only in LS174T WT cells (Figure 2C and Supplementary Figure S2B). All the effects at the level of lipid hydroperoxides or viability were completely reverted by the addition of ferrostatin-1, suggesting no off-target effects of the drug. Similarly, when the viability of WT and FSP1-KO cells was investigated under cysteine-deprivation conditions, no difference between them was detected (Supplementary Figure S2D), except for the fact that ferrostatin-1 completely reverted mild effect of cysteine-starvation observed in FSP1-KO, but not in WT cells.

To understand the specific ability of LS174T to resist ferroptosis induction after pharmacological/genetic inhibition of both major ferroptosis-preventing pathways we measured the cellular level of ROS, the initiator of lipid hydroperoxide formation. The data showed that ROS content changed in the same way as the level of lipid hydroperoxides (Figure 2D and Supplementary Figure S2E). FSP1-KO cells were not characterized by higher ROS content compared to WT cells (Figure 2D and Supplementary Figure S2E, black bars). However, after 4 h of RSL3 treatment, there was an increase in ROS accumulation in both cell lines, which was more prominent in the FSP1-KO cells. This effect was completely absent after 24 h of treatment or in the presence of ferrostatin-1 (Figure 2D and Supplementary Figure S2E, gray bars). Results presented here clearly point toward efficient adaptive mechanism(s) employed by both WT and FSP1-KO cells after 24 h of RSL3 treatment.

### 3.3. Rewiring Cellular Metabolism toward OXPHOS Is Not Enough to Sensitize Cells to Ferroptosis

Considering that simultaneous targeting of an alternative ferroptosis-preventing antioxidant pathway did not result in the expected increase in sensitivity of LS174T cells to GPX4 inhibition, we wondered if the opposite approach would be more efficient. Namely, instead of suppressing antioxidative capacity, we aimed to increase ROS content within the cell and investigate if this strategy can sensitize LS174T cells toward GPX4 inhibition. Considering that mitochondria, i.e., mitochondrial OXPHOS, represent the major source of cellular ROS, we wanted to investigate if the switch toward oxidative metabolism would lower the threshold necessary for ferroptosis induction. Our previous studies dealing with the Warburg effect [24] in aggressive tumors (colorectal adenocarcinoma, among them) revealed that an efficient way to switch toward oxidative metabolism is by genetically deleting both isoforms A and B of LDH (here referred to as LDH-KO). In the mentioned study of [24], our team generated LDH-KO in colorectal adenocarcinoma (LS174T) and melanoma (B16-F10), which we used in the present study.

**Figure 3:**
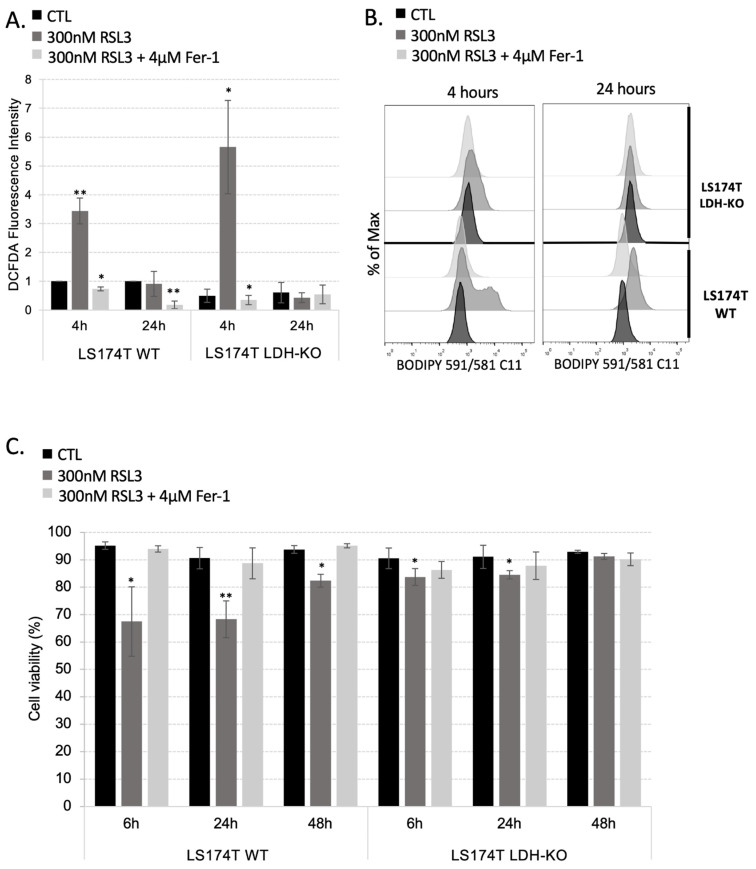
Metabolism rewiring to OXPHOS renders LS174T cells more resistant toward RSL3-induced ferroptosis. (**A**) Intracellular ROS level was measured by DCFDA staining (see Section 2) in LS174T WT and LDH-KO 4 h and 24 h after seeding in media supplemented or not with 300 nM RSL3 and 4 µM Fer-1. Intracellular ROS levels are presented as fold of change (mean ± SEM, *n* = 3). * *p* < 0.05, ** *p* < 0.01, comparison with corresponding cell line in control conditions. (**B**) Lipid hydroperoxide content was measured in LS174T WT and LDH-KO cells 4 h and 24 h upon seeding in media supplemented or not with 300 nM RSL3 and 4 µM Fer-1. Presented histograms are representative of three independent experiments. (**C**) Cell viability was measured in LS174T WT and LDH-KO cells using PI staining method (see Section 2). Cells were seeded in media with or without 300 nM RSL3, supplemented or not with 4 µM Fer-1. Cell viability was analyzed after 6 h, 24 h and 48 h. The results are presented as mean ± SEM, *n* = 3. * *p* < 0.05, ** *p* < 0.01, comparison with corresponding cell line in control conditions.

Data obtained here showed that, although LDH-KO cells do not show higher baseline ROS levels in comparison with their WT counterparts, the inhibition of the GPX4 enzyme in these cells induced a significant ROS burst after just 4 h of treatment (Figure 3A). However, just as in the case of WT and FSP1-KO cells, the ROS level in LDH-KO returned to control after 24 h of RSL3 treatment or by ferrostatin-1, suggesting the adaptation of the cells to the GPX4 inhibition. Surprisingly, the level of lipid hydroperoxides in LDH-KO was only slightly increased after 4 h and completely restituted after 24 h of RSL3 treatment (Figure 3B). Furthermore, the LDH-KO cells looked healthier than the WT cells after 4 h, 6 h and 24 h of treatment, with almost unchanged viability at any investigated time point of the treatment (Figure 3C). Cell death in LDH-KO was also analyzed after 48 h of cystine/cysteine starvation and showed higher resistance to ferroptosis when compared to the WT cells (Supplementary Figure S3).

Considering the great resistance LS174T LDH-KO cells showed toward ferroptosis-induction either by GPX4 inhibition or cysteine starvation, we were curious to investigate if the “Warburg-null” melanoma cell line, we previously obtained [24], shows the same phenotype. Namely, according to obtained data the melanoma cell line B16-F10, shows much higher sensitivity to ferroptosis; 48-h culturing in media without cysteine resulted in the nearly complete death of the B16-F10 cells (Supplementary Figure S4A). Similarly, according to clonogenicity and cell viability assay, 300 nM RSL3 (previously used without effect on LS174T cells) led to 70% of cell death and almost no visible clones in the clonogenicity test (Supplementary Figure S4B,C). The concentration of RSL3 that show no effect on the viability of these cells was 10× lower (30 nM), and thus this concentration was used further in the study. Interestingly, although 30 nM RSL3 did not show any effect on cell death after 48 h (Supplementary Figure S4C), our data showed that it did induce an initial burst in lipid hydroperoxides in B16-F10 WT cells, which was restituted with ferrostatin-1 or within one day of treatment (Supplementary Figure S4D). However, like in LS174T cells, the genetic deletion of both LDHA and B in this cell line showed no visible increase in lipid hydroperoxide accumulation in the control or RSL3-treated conditions (Supplementary Figure S4D), the cells looked healthy with no detectable cell death after 24 or 48 h of treatment (Supplementary Figure S4E).

It is worth noting here that we also generated B16-F10 FSP1-KO (Supplementary Figure S5A) to investigate if the same adaptation as in LS174T will happen if FSP1 was genetically deleted. However, according to our data, genetic deletion of FSP1 in this cell line led to increased sensitivity to RSL3 treatment visible both on the level of lipid hydroperoxide content (Supplementary Figure S5B) and viability (Supplementary Figure S5C), which clearly points out the unique nature of ferroptosis-resistance of CRC cells. Results obtained with B16-F10 WT, FSP1-KO and LDH-KO are summarized in the clonogenicity assay presented in Supplementary Figure S6.

### 3.4. Combination of Metabolic Rewiring to OXPHOS and Inhibition of Both Ferroptosis-Preventing Pathways Is Necessary to Sensitize CRC to Ferroptosis

**Figure 4:**
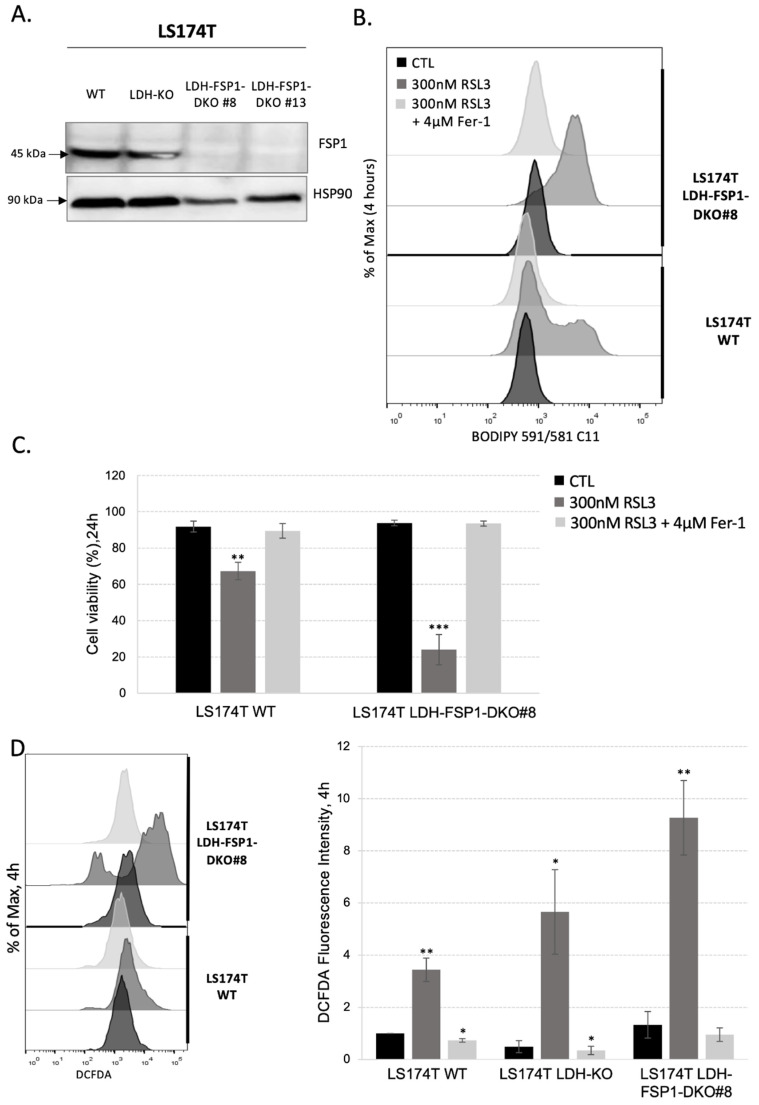
Suppression of the Warburg effect is necessary to induce ferroptosis in LS174T cells with FSP1-KO genetic background. (**A**) The protein expression of FSP1 was analyzed in LS174T WT, LDH-KO and two independent LDH-FSP1-DKO (#8 and #13) clones. Three independent experiments were performed, and representative blots are shown. (**B**) Lipid hydroperoxide content was measured in LS174T WT and LDH-FSP1-DKO #8 cells 4 h upon seeding in media supplemented or not with 300 nM RSL3 and 4 µM Fer-1. Presented histograms are representative of three independent experiments. (**C**) Cell viability was measured in LS174T WT and LDH-FSP1-DKO #8 cells using PI staining method (see Section 2). Cells were seeded in media supplemented or not with 300 nM RSL3, in the presence or not of 4 µM Fer-1. Cell viability was analyzed after 24 h. The results are presented as mean ± SEM, *n* = 3. ** *p* < 0.01, *** *p* < 0.001, comparison with corresponding cell line in control conditions. (**D**) Intracellular ROS level was measured by DCFDA staining (see Section 2) in LS174T WT and LDH-FSP1-DKO #8 4 h after seeding in media supplemented or not with 300 nM RSL3, in the presence or not of 4 µM Fer-1. Intracellular ROS levels are presented as representative histograms and as a fold of change (mean ± SEM, *n* = 3). * *p* < 0.05, ** *p* < 0.01, comparison with corresponding cell line in control conditions.

To investigate how the combination of OXPHOS metabolism rewiring and inhibition of the two major ferroptosis-preventing pathways, GSH-GPX4 and FSP1-CoQ10 pathways, could sensitize LS174T cells to ferroptosis, we generated an FSP1 genomic deletion using the CRISPR-Cas9 technique in the cells with an LDH-KO background. Analysis of genomic DNA and sequencing of the CRISPR-targeted site demonstrated disruptive mutations in FSP1 that caused the lack of corresponding protein expression in LDH-FSP1-DKO clones of both cell lines, as shown by Western blot analysis (Figure 4A). Two independent clones were analyzed and showed the same results, to simplify one of these clones is represented in Supplementary Figure S7.

We examined lipid hydroperoxide levels in LS174T WT and LDH-FSP1-DKO cells (Figure 4B and Supplementary Figure S7A) and we correlated these results to cell death analysis (Figure 4C and Supplementary Figure S7B). The combination of FSP1 deletion and metabolism shift toward OXPHOS did not induce a significant increase in lipid hydroperoxide content compared with WT cells (Figure 4B and Supplementary Figure S7A, black lines) or cell death (Figure 4C and Supplementary Figure S7B, black bars), meaning that the combination of these two modalities was not sufficient to induce ferroptosis. However, after 4 h of RSL3 treatment, a significant increase in lipid hydroperoxide content was observed in LDH-FSP1-DKO when compared with WT, which was completely rescued by the addition of ferrostatin-1 in each case. An analysis of cell death after 24 h of RSL3 treatment showed that this high increase in lipid hydroperoxide content consequently caused a 90% decrease in LDH-FSP1-DKO viability (Figure 4C and Supplementary Figure S7B). This loss of viability was also completely rescued by the addition of ferrostatin-1, suggesting a ferroptosis-specific cellular response. Considering the high level of cell death in this cell line, analysis of lipid hydroperoxides after 24 h of RSL3 treatment could not be performed. Nearly 100% cell death was also observed after 48 h of cystine/cysteine starvation, which is in sharp contrast with only approximately 30% of cell death in WT in these conditions (Supplementary Figure S7C).

The level of ROS content was consistent with the level of lipid hydroperoxides. After 4 h of RSL3 treatment, we observed a high increase in cellular ROS content in LDH-FSP1-DKO compared with WT cells (Figure 4D and Supplementary Figure S7D). Quantification of these results showed that the ROS level in LDH-FSP1-DKO was even higher than the ROS level in LDH-KO (Figure 4D).

**Figure 5:**
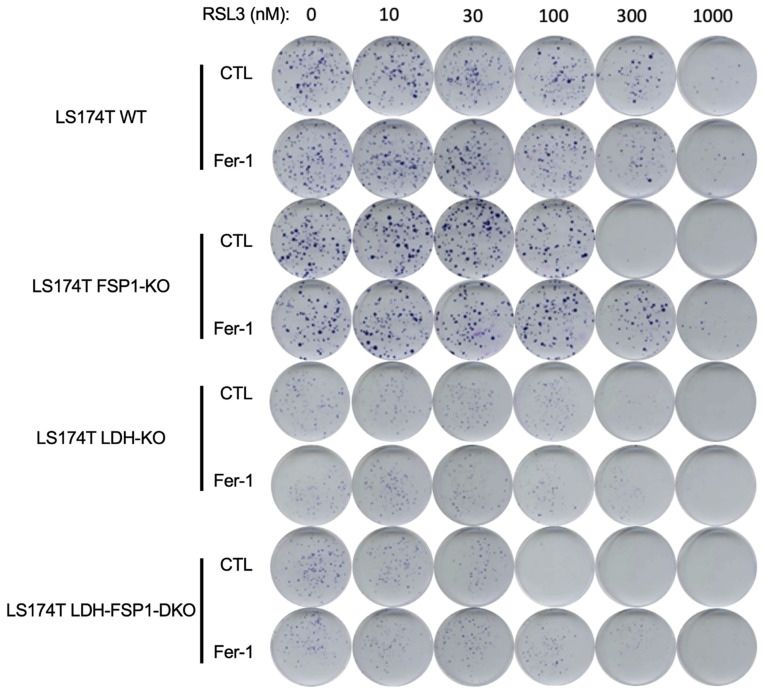
The effect of pharmacological inhibition of GPX4 in LS174T cells with WT, LDH-KO, FSP1-KO or LDH-FSP1-DKO genetic background. Cells were cultivated 14 days in DMEM media supplemented with increasing concentration of RSL3 (0, 10, 30,100, 300, 1000 nM) in the presence or not of 4 µM Fer-1. At the end of the experiment, colonies were colored for visualization using Giemsa. Representative images are shown.

These data indicate that a deletion of FSP1 in LS174T cells with an LDH-KO background was the only way to increase sensitivity to GPX4-inhibition-induced ferroptosis in LS174T. Moreover, LDH-FSP1-DKO were unable to restore their cellular ROS and/or lipid hydroperoxides levels as in WT, LDH-KO and FSP1-KO cells after 24 h of RSL3 treatment (Figure 2B,D and Figure 3A). Several explanations for this are conceivable. After 24 h of RSL3 treatment, FSP1-KO cells could potentially switch to glycolytic metabolism; the rescue in LDH-KO cells could be due to the activity of the FSP1-CoQ10 pathway. These data were summarized in a clonogenicity assay on LS174T WT, LDH-KO, FSP1-KO and LDH-FSP1-DKO cells seeded with increasing concentrations of RSL3 in the presence or not of ferrostatin-1 (Figure 5).

## 4. Discussion

This year marks 10 years since Stockwell’s team officially coined the term “ferroptosis” for a very specific, unprogrammed type of cell death that is caused by the uncontrolled accumulation of lipid hydroperoxides in the plasma membrane of the cell [1]. The initial hypothesis pointing toward the irreplaceable role of GPX4 in lipid hydroperoxide removal, and thus, ferroptosis prevention, has been to a certain degree, updated with the discovery of alternative lipid peroxide scavenging pathways, such as coenzyme Q-ferroptosis suppressor protein 1 (CoQ-FSP1) [14,15], as well as dihydrofolate reductase-tetrahydrobiopterin (DHFR-BH4) [25,26]. Nonetheless, it would be unrealistic to assume that this update is the final one. In the present study, we investigated cellular contexts which govern the resistance or sensitivity to ferroptosis.

Colorectal adenocarcinoma (CRCs) cells are known as the second deadliest cancer worldwide [27] notoriously resistant to the conventionally used chemotherapeutics (reviewed in [28]). Furthermore, our data showed that this type of cancer shows great resistance to ferroptosis induction either by cysteine starvation, GPX4 inhibition, or FSP1 inhibition (Figure 1A–C and Supplementary Figure S1). These results are in great accordance with the previous studies [25,29], which reported similar resistance of the CRC cells to ferroptosis induction either by erastin (inhibitor of cystine uptake) or RSL3 (inhibitor of GPX4). Authors of the mentioned studies, however, focused exclusively on the DHFR-BH4 pathway as an alternative to the canonical xCT-GSH-GPX4 axis, claiming its pivotal role in observed resistance. On the other hand, the high protein content of FSP1 in all investigated CRC cell lines (LS174T, HCT 15, HCT 116 and SW480) (Figure 1D), led us to hypothesize the potential role of this alternative pathway in the ferroptosis resistance. Interestingly, although pharmacological inhibition of FSP1 (by iFSP1), as in the case of GPX4 inhibition (by RSL3), did not induce cell death, a combination of iFSP1 and RSL3 compromised the survival of the CRC cells, with approximately 50% cell death after 24 h of treatment (Supplementary Figure S1). On the contrary, in the study of Hu and colleagues, the depletion of the BH4 did not synergize with RSL3 but did with erastin treatment [29]. Synergy with erastin is very difficult to interpret considering that erastin can affect, not only GPX4 activity through the GSH content, but also endoplasmic reticulum stress [30,31], and potentially disrupts the CoQ-FSP1 axis considering that a large portion of cysteine is incorporate in the Coenzyme A (via pantothenate pathway), which is a precursor for CoQ synthesis [32]. Thus, it is impossible to unequivocally conclude that FSP1 did not play an important role in the mentioned study as well.

Although selective and efficient pharmacological means are always the major goal in a clinical setting, the genetic approach still represents an unbeatable tool for the fundamental science and understanding of the role and dispensability of individual cellular players. Therefore, we obtained FSP1-KO in the LS174T cell line (Figure 2A), which showed the highest expression of anti-ferroptotic markers (Figure 1D). As expected, genetic deletion of FSP1 alone did not induce accumulation of lipid hydroperoxides, nor cell death after 4/6 h, 24 h or 48 h after seeding (Figure 2B,C). Surprisingly, however, RSL3 treatment or cysteine starvation did not have the phenocopied effects observed with iFSP1 in FSP1-KO cells (Figure 2B–D, Supplementary Figures S1 and S2A,B). A potential explanation for this kind of discrepancy is always off-targets of the drug. However, the use of iFSP1 and RSL3, which induced 50% of cell death in WT cells, on FSP1-KO cells did not show any effect (Supplementary Figure S2C), suggesting potential resistance mechanism(s) developed in the FSP1-KO cells, a phenomenon known as a “genetic compensation” (reviewed in [33]). This is of utmost importance from the translational stand of point of science, considering that acute effects of the drug or genetic silencing are very frequently taken for granted, leading to “unexpected” resistant mechanisms when chronic treatment (or knockout, for that matter) is applied [34].

When we thought about potential resistance mechanisms employed by the FSP1-KO, one important aspect of their behavior toward RSL3 treatment was taken into consideration, the kinetics of lipid hydroperoxide accumulation. Namely, for both WT and FSP1-KO cells, very short treatment (4 h) with RSL3 induced accumulation of lipid hydroperoxides, and more prominently in FSP1-KO than in WT cells (Figure 2B and Supplementary Figure S2A, panels on the left). Nonetheless, after 24 h almost no increase in the BODIPY C11 staining was detectable in both cell lines. A similar trend was observed with the cellular content of ROS (Figure 2D and Supplementary Figure S2E, panels on the right). These kinetics indicate that adaptation might be more phenotypic and less genetic. Considering that the level of cellular ROS decreased with time, a logical hypothesis that was imposed was—is it possible that CRC suppressed internal production of ROS by switching from an oxidative to glycolytic mode in response to compromised antioxidative defense? Oxidative phosphorylation (OXPHOS) within mitochondria, is the major source of ROS. The importance of mitochondrial metabolism for redox homeostasis is reflected in the fact that the major regulator of redox homeostasis, NRF2, regulates many antioxidant enzymes, also glutaminase (GLS) isoenzymes. GLS is a group of enzymes, whose expression is very frequently alleviated in cancer, which regulates cellular redox homeostasis by fueling the GSH biosynthetic pathway, but also oxidative metabolism by fueling TCA [35]. A recent study by [36] showed that the genetic silencing of GLS2 leads to decreased sensitivity of the hepatocellular carcinoma cells to ferroptosis. This is in great accordance with the previous work of Jiang’s team who showed that oxidative metabolism (TCA cycle and OXPHOS) plays a pivotal role in promoting ferroptosis induced by cysteine deprivation [37,38]. The authors showed that inhibiting the TCA cycle could suppress the mitochondrial membrane depolarization, and thus, ferroptosis, in tumor cells [38]. These results are in great accordance with the previously shown correlation between the Warburg effect and drug resistance in CRC (reviewed in [39]). Thus, targeting the Warburg effect in combination with chemotherapy proved to be an efficient way to increase the sensitivity of CRC [40]. Considering our previous extensive work on the Warburg effect in cancer cells [20,24,41,42,43,44,44,45,46,47,48,49,50,51,52,53], we decided to investigate this hypothesis in more detail. According to our previous study, the most efficient way to obtain Warburg-incompetent cells is to genetically invalidate both isoforms of lactate dehydrogenase (LDH-KO). Considering that we previously obtained LDH-KO in the LS174T cell line [24], we first characterized the sensitivity of these cells to ferroptosis induction by GPX4 inhibition, and then we used these cells to generate LDH-FSP1-DKO. Surprisingly, although LDH-KO cells showed an initial burst in cellular ROS content upon RSL3 treatment (Figure 3A), they showed no signs of ferroptotic cell death (lipid hydroperoxide accumulation, cell death) within 48 h time frame (Figure 3B,C). Even more, these cells showed lower initial content of lipid hydroperoxides and looked much healthier under a microscope upon RSL3 exposure in comparison with their WT counterparts (Figure 3B). Considering that we had at our disposal another Warburg-incompetent cell line, mouse melanoma B16-F10 [24], we investigated if the phenomenon is universal. Indeed, although the B16-F10 cell line showed much higher sensitivity to GPX4 inhibition (Supplementary Figure S4A–C), the genetic deletion of both LDH isoforms made these cells more resistant to ferroptosis, as in the case of LS174T LDH-KO cells (Supplementary Figure S4D,E). It is worth noting here that we also obtained B16-F10 FSP1-KO cells to investigate the universality of the “genetic compensation” observed in their LS174T counterparts, but the same was not detected in this cell line (Supplementary Figures S5A–C and S6), suggesting that LS174T FSP1-KO cells have a higher level of phenotypic flexibility.

A potential explanation regarding the resistance of LDH-KO to ferroptosis could be found in the slower growth rate of these cells (Supplementary Figure S6), considering that the growth rate determines the metabolic rate and the latter intracellular ROS generation. Thus, to investigate if these cells are more sensitive to compromised antioxidant defense, as well as if we can tackle the resistance of the FSP1-KO cells, we generated LS174T LDH-FSP1-DKO cells (Figure 4A). Indeed, resistance observed in the FSP1-KO cells of WT background was completely abolished. The LDH-FSP1-DKO cells showed much higher sensitivity to RSL3 treatment (Figure 4B–D and Figure 5), which is in great accordance with the recent study of Ludikhuize and colleagues [40]; 24-h treatment induced 80% cell death, which is higher than previously observed with iFSP1 + RSL3 in WT cells (Supplementary Figure S1).

## 5. Conclusions

In conclusion, our results showed that targeting individual anti-ferroptotic pathways may not be sufficient to induce cell death in CRC cells or can potentially lead to the adaptation of the cells to the treatment. Therefore, when it comes to highly resistant cells it is necessary to bear in mind the cellular context and phenotypic plasticity of a particular cancer type. In the case of CRC cells, it seems that the inhibition of GSH-GPX4 and CoQ-FSP1 axes should be coupled with other metabolic modulators, such as inhibitors of glycolysis/Warburg effect.

## Data Availability

The data presented in this study are available in the article and Supplementary Materials.

## Supplementary Materials

The following supporting information can be downloaded at: https://www.mdpi.com/article/10.3390/antiox11122412/s1, Figure S1: The sensitivity of the CRC cells to cysteine starvation; Figure S2: Characterization of the FSP1-KO#6; Figure S3: The sensitivity of the LS174T WT and LDH-KO to cysteine starvation; Figure S4: Ferroptosis in B16-F10; Figure S5: B16-F10 FSP1-KO are sensitive to GPX4 inhibition; Figure S6: Clonal growth of B16-F10 WT, FSP1-KO and LDH-KO cells; Figure S7: Metabolic rewiring toward OXPHOS disrupts intrinsic resistance to ferroptosis of the CRC cells.
