# Supplementary material for: Metabolic Rewiring toward Oxidative Phosphorylation Disrupts Intrinsic Resistance to Ferroptosis of the Colon Adenocarcinoma Cells
Source: Antioxidants (Basel). 2022 Dec 6;11(12):2412. doi: 10.3390/antiox11122412 (PMC9774558; doi:10.3390/antiox11122412)
Supplement: Supplementary file 1 [file antioxidants-11-02412-s001.zip › antioxidants-2053170-supplementary.pdf]

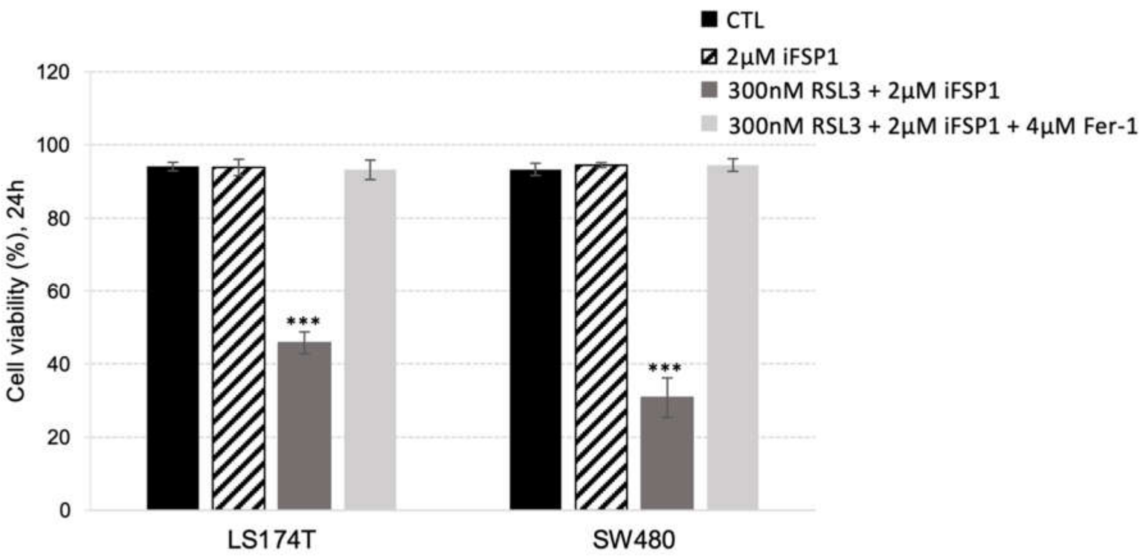

Supplementary Figure S1

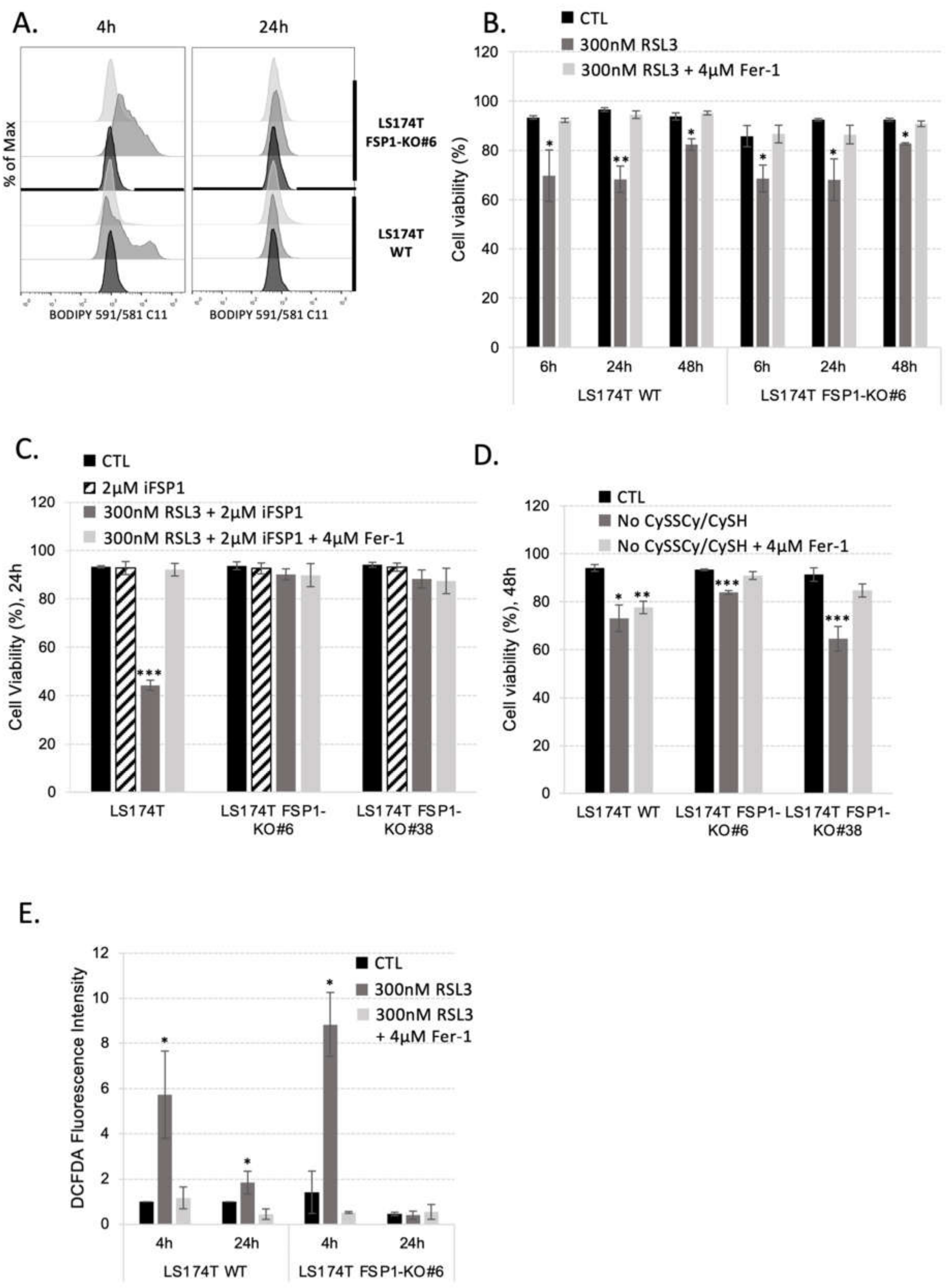

Supplementary Figure S2

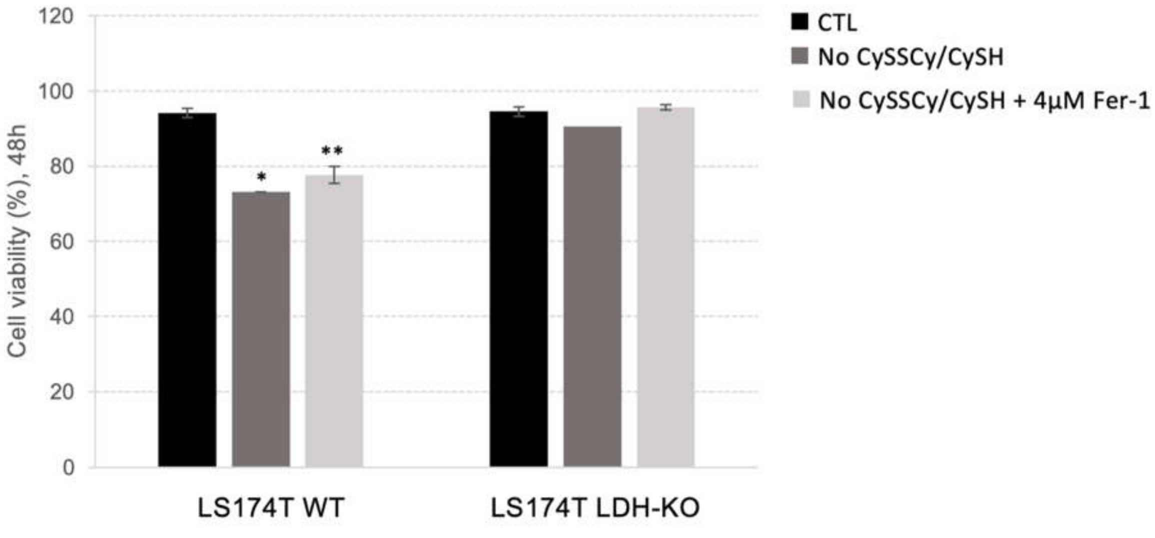

Supplementary Figure S3

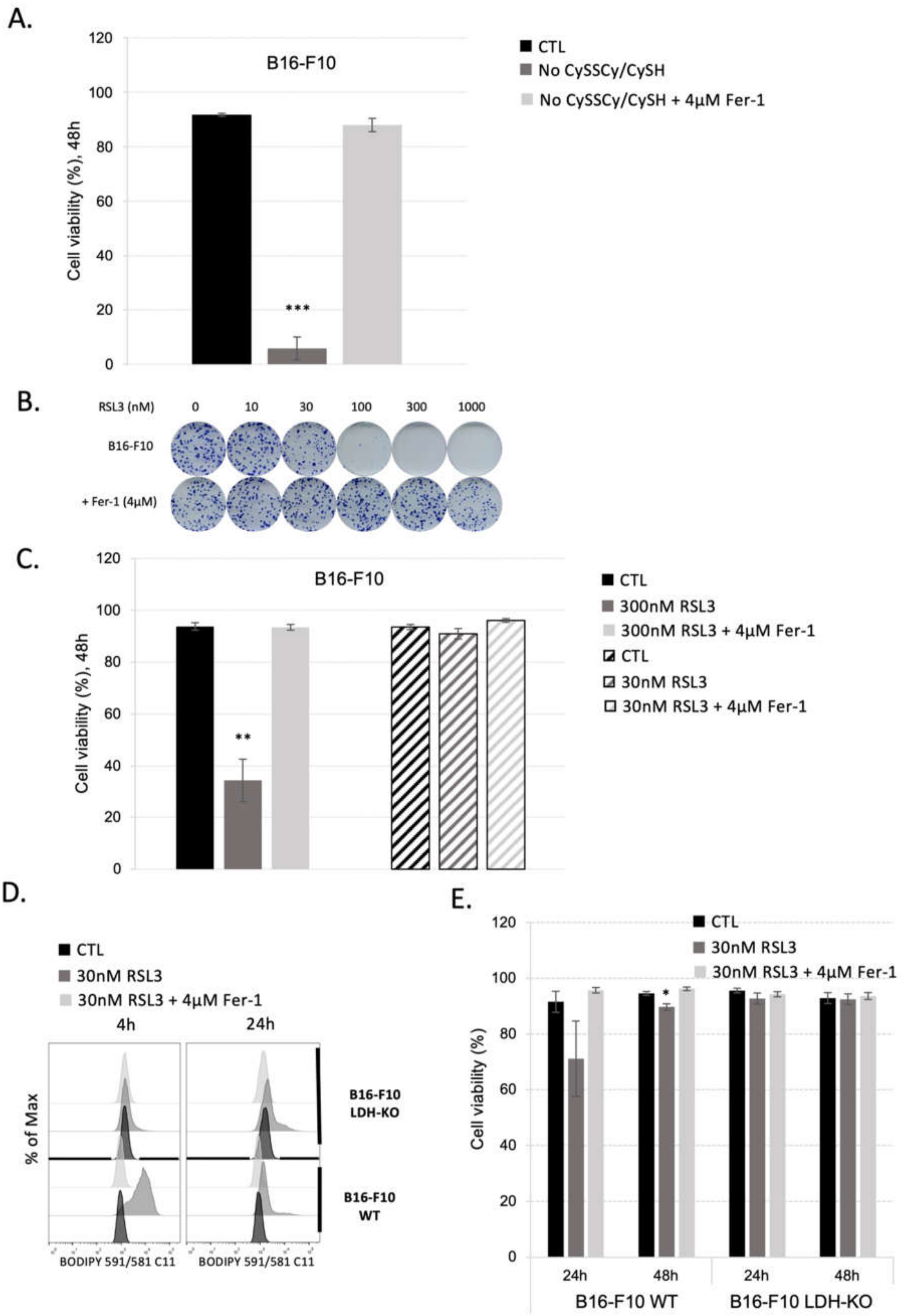

Supplementary Figure S4

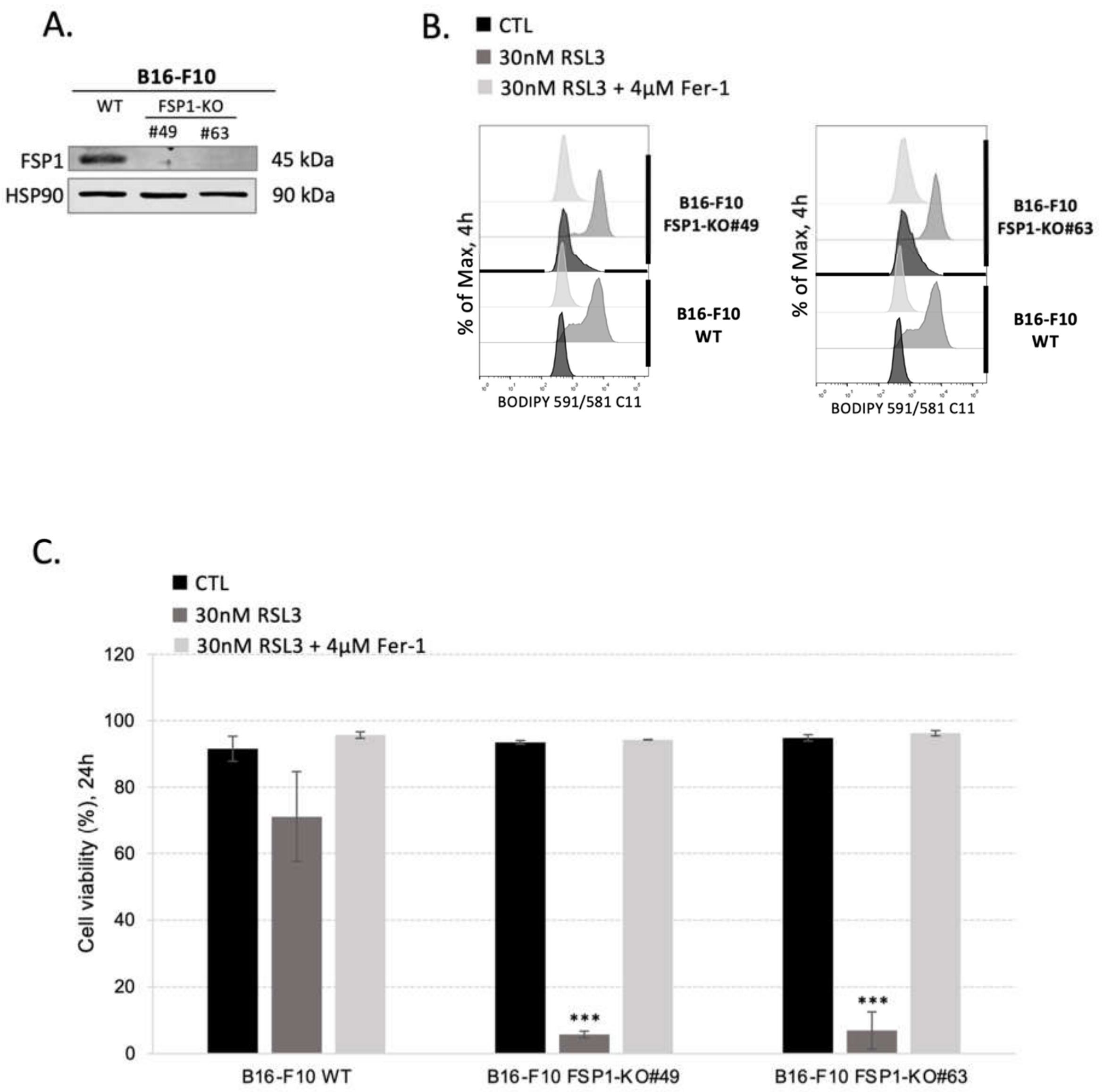

Supplementary Figure S5

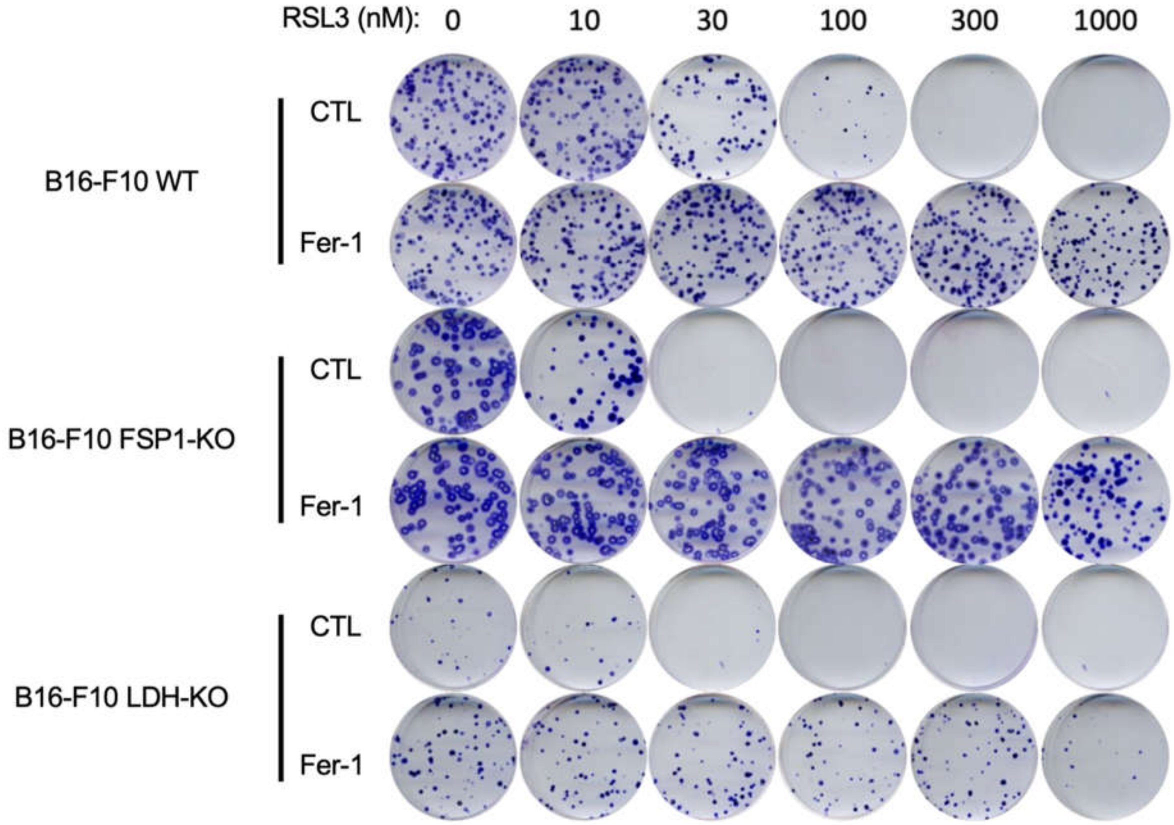

Supplementary Figure S6

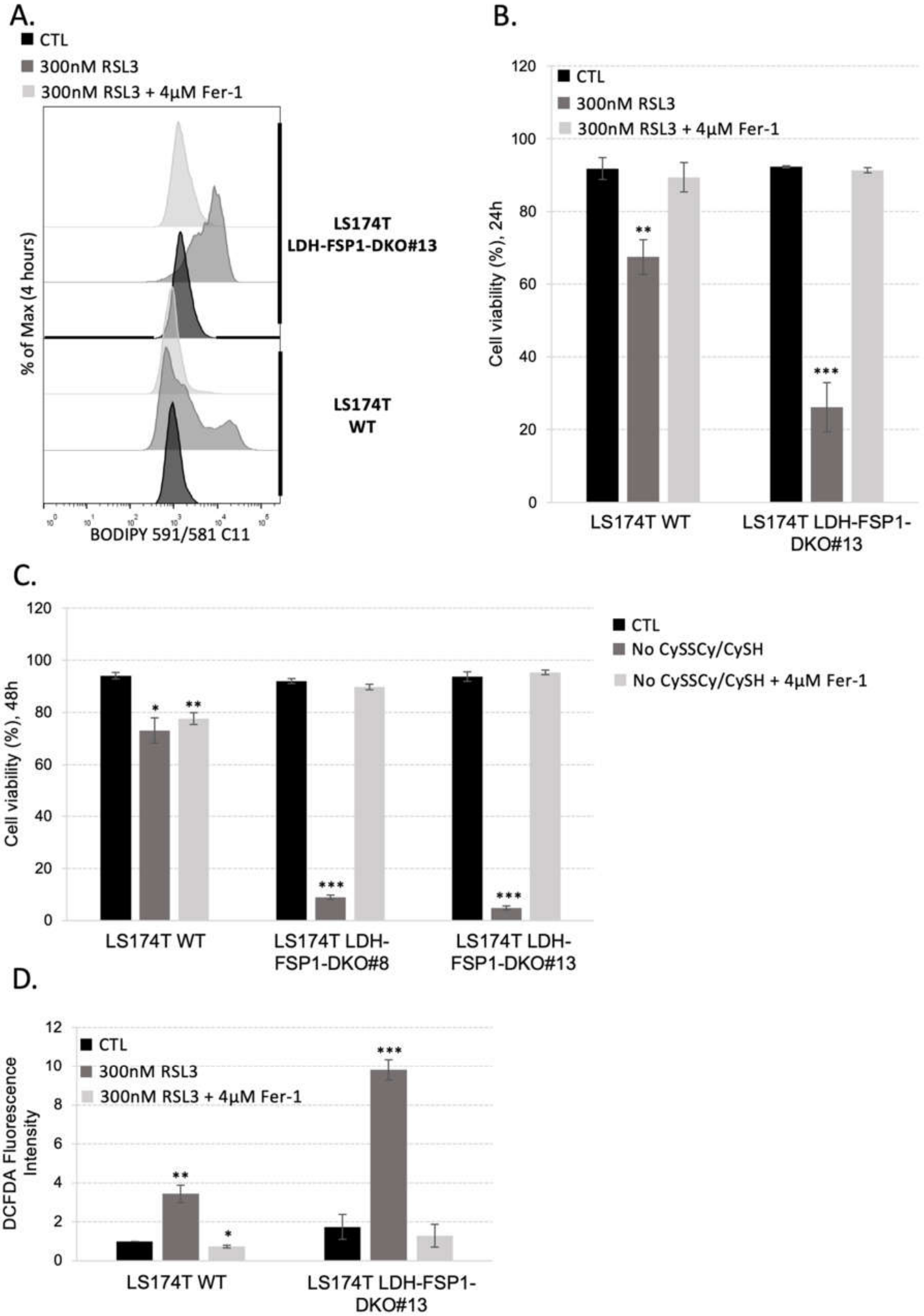

Supplementary Figure S7

SUPPLEMENTARY FIGURE LEGENDS:

Supplementary Figure S1.

The sensitivity of the CRC cells to cysteine starvation. Cell viability was measured in LS174T and SW480 cells using PI staining method (see Material & Methods). Cells were seeded in media supplemented or not with 2μM iFSP1, 300nM RSL3, in the presence or not of 4μM Fer-1. Cell viability was analyzed after 24h and the results are presented as mean ± SEM, n=3. ***$p<0.001$, *comparison with corresponding cell line in control conditions.

Supplementary Figure S2.

Characterization of the FSP1-KO#6.

A. Lipid hydroperoxide content was measured in LS174T WT and FSP1-KO#6 cells 4h and 24h upon seeding in media supplemented or not with 300nM RSL3, in the presence or not of 4μM Fer-1. Presented histograms are representative of three independent experiments.
B. Cell viability was measured in LS174T WT and FSP1-KO#6 cells using PI staining method (see Material & Methods). Cells were seeded in media supplemented or not with 300nM RSL3, in the presence or not of 4μM Fer-1. Cell viability was analyzed after 6h, 24h and 48h. The results are presented as mean ± SEM, n=3. *$p<0.05$, **$p<0.01$, *comparison with corresponding cell line in control conditions.
C. Cell viability was measured in LS174T WT and FSP1-KO#6 cells using PI staining method (see Material & Methods). Cells were seeded in media supplemented or not with 2μM iFSP1, 300nM RSL3, in the presence or not of 4μM Fer-1. Cell viability was analyzed after 24h. The results are presented as mean ± SEM, n=3. ***$p<0.001$, *comparison with corresponding cell line in control conditions.
D. LS174T WT and FSP1-KO#6 and #38 cells were seeded in media containing or not cyst(e)ine, supplemented or not with 4μM Fer-1. Cell viability was analyzed after 48 hours. The results are presented as mean ± SEM, n=3. *$p<0.05$, **$p<0.01$, ***$p<0.001$, *comparison with corresponding cell line in control conditions.
E. Intracellular ROS level was measured by DCFDA staining (see Material & Methods) in LS174T WT and FSP1-KO#6 4h and 24h after seeding in media supplemented or not with 300nM RSL3, in the presence or not of 4μM Fer-1. Intracellular ROS level are presented as a fold of change (mean ± SEM, n=3). *$p<0.05$, *comparison with corresponding cell line in control conditions.

Supplementary Figure S3.

The sensitivity of the LS174T WT and LDH-KO to cysteine starvation.

LS174T WT and LDH-KO cells were seeded in media containing or not cyst(e)ine, supplemented or not with 4μM Fer-1. Cell viability was analyzed after 48 hours. The results are presented as mean ± SEM, n=3. *$p<0.05$, **$p<0.01$, *comparison with corresponding cell line in control conditions.

Supplementary Figure S4.

Ferroptosis in B16-F10.

A. B16-F10 cells were seeded in the media supplemented or not with cyst(e)ine, in the presence or not of 4μM Fer-1. After 48h cell death was analyzed using PI staining. ***$p<0.001$, *comparison with the control.
B. B16-F10 cells were cultivated in DMEM media supplemented with increasing concentration of GPX4 inhibitor - RSL3 (0, 10, 30, 100, 300, 1000 nM), supplemented or not with 4μM Ferrostatin-1 (Fer-1). After 7 days colonies were colored for visualization using Giemsa. Representative images are shown.

C. B16-F10 cells were seeded in the media supplemented or not with 300nM or 30nM RSL3, in the presence or not of 4μM Fer-1. After 48h cell death was analyzed. **p<0.01, *comparison with the corresponding control.
D. Lipid hydroperoxide content was measured in B16-F10 WT and LDH-KO 4h and 24h upon seeding in media supplemented or not with 30nM RSL3 and 4μM Fer-1. Presented histograms are representative of three independent experiments.
E. Cell viability was measured in WT and LDH-KO B16-F10 using PI staining method (see Material & Methods). Cells were seeded in media with 30nM RSL3, supplemented or not with 4μM Fer-1. Cell viability was analyzed after 24h and 48h. The results are presented as mean ± SEM, n=3. *p<0.05, *comparison with corresponding cell line in control conditions.

Supplementary Figure S5.
B16-F10 FSP1-KO are sensitive to GPX4 inhibition.
A. The expression of FSP1 was analyzed in B16-F10 WT and two independent FSP1-KO (#49 and #63). Three independent experiments were performed, and representative blots are shown.
B. Lipid hydroperoxide content was measured in B16-F10 WT and two independent FSP1-KO (#49 and #63) 4h upon seeding in media supplemented or not with 30nM RSL3, in the presence or not of 4μM Fer-1. Presented histograms are representative of three independent experiments.
C. Cell viability was measured in B16-F10 WT and two independent FSP1-KO (#49 and #63) using PI staining method (see Material & Methods). Cells were seeded in media with or not 30nM, in the presence or not of 4μM Fer-1. Cell viability was analyzed after 24 hours. The results are presented as mean ± SEM, n=3. ***p<0.001, *comparison with corresponding cell line in control conditions.

Supplementary Figure S6.
Clonal growth of B16-F10 WT, FSP1-KO and LDH-KO cells. Cells were cultivated 7 days in DMEM media supplemented with increasing concentration of RSL3 (0, 10, 30, 100, 300, 1000nM), in the presence or not of 4μM Fer-1. At the end of experiment clones were colored for visualization using Giemsa. Representative images are shown.

Supplementary Figure S7.
Metabolic rewiring toward OXPHOS disrupts intrinsic resistance to ferroptosis of the CRC cells.
A. Lipid hydroperoxide content was measured in LS174T WT and LDH-FSP1-DKO#13 cells 4h upon seeding in media supplemented or not with 300nM RSL3, in the presence or not of 4μM Fer-1. Presented histograms are representative of three independent experiments.
B. LS174T WT and LDH-FSP1-DKO#13 cells were seeded in media supplemented or not with 300nM RSL3, in the presence or not of 4μM Fer-1. Cell viability was analyzed after 24 hours. The results are presented as mean ± SEM, n=3. **p<0.01, ***p<0.001, *comparison with corresponding cell line in control conditions.
C. LS174T WT and two independent LDH-FSP1-DKO clones (#8 and #13) were seeded in media containing or not cyst(e)ine and supplemented or not with 4μM Fer-1. Cell viability was analyzed after 48 hours. The results are presented as mean ± SEM, n=3. *p<0.05, **p<0.01, ***p<0.001, *comparison with corresponding cell line in control conditions.
D. Intracellular ROS level was measured in LS174T WT and FSP1-KO#13 4h and 24h after seeding in media supplemented or not with 300nM RSL3, in the presence or not of 4μM Fer-1. Intracellular ROS level are presented as a fold of change (mean ± SEM, n=3). *p<0.05, **p<0.01, ***p<0.001, *comparison with corresponding cell line in control conditions.
